# *Caenorhabditis Elegans* as a Model for Environmental Epigenetics

**DOI:** 10.1007/s40572-025-00472-z

**Published:** 2025-01-20

**Authors:** Adam Filipowicz, Patrick Allard

**Affiliations:** 1 Institute for Society and Genetics, University of California, Boyer Hall, Room 332, 611 Charles E Young Dr E., UCLA, Los Angeles, CA 90095 USA; 2 Environmental and Molecular Toxicology Program, University of California, Los Angeles, USA

**Keywords:** Epigenetics, Transgenerational inheritance, Environmental toxicology, Developmental origins of health and disease, Histone post-translational modifications, Non-coding RNAs

## Abstract

**Purpose of Review:**

The burgeoning field of environmental epigenetics has revealed the malleability of the epigenome and uncovered numerous instances of its sensitivity to environmental influences; however, pinpointing specific mechanisms that tie together environmental triggers, epigenetic pathways, and organismal responses has proven difficult. This article describes how *Caenorhabditis elegans* can fill this gap, serving as a useful model for the discovery of molecular epigenetic mechanisms that are conserved in humans.

**Recent Findings:**

Recent results show that environmental stressors such as methylmercury, arsenite, starvation, heat, bacterial infection, and mitochondrial inhibitors can all have profound effects on the epigenome, with some insults showing epigenetic and organismal effects for multiple generations. In some cases, the pathways connecting the stressor to epigenetic pathways and organismal responses have been elucidated. For example, a small RNA from the bacterial pathogen *Pseudomonas aeruginosa* induces transgenerational learned avoidance by activating the RNA interference PIWI-interacting RNA pathways across generations to downregulate, via *Cer1* retrotransposon particles and histone methylation, *maco-1,* a gene that functions in sensory neurons to regulate chemotaxis. Mitochondrial inhibitors seem to have a profound effect on both the DNA methylation mark 6mA and histone methylation, and may act within mitochondrial DNA (mtDNA) to regulate mitochondrial stress response genes. Transgenerational transcriptional responses to alcohol have also been worked out at the single-nucleus resolution in *C. elegans*, demonstrating its utility when combined with modern sequencing technologies.

**Summary:**

These recent studies highlight how *C. elegans* can serve as a bridge between biochemical in vitro experiments and the more associative findings of epidemiological studies in humans to unveil possible mechanisms of environmental influence on the epigenome. The nematode is particularly well-suited to transgenerational experiments thanks to its rapid generation time and ability to self-fertilize. These studies have revealed connections between the various epigenetic mechanisms, and so studies in *C. elegans* that take advantage of recent advancements in sequencing technologies, including single-cell techniques, to gain unprecedented resolution of the whole epigenome across development and generations will be critical.

## Introduction

Although the term epigenetics was first used by Conrad Waddington in the 1940s to describe how changes in the genotype give rise to distinct phenotypes [1, 2], the more modern field of epigenetics is broadly concerned with changes in gene expression and phenotypes in the absence of changes to DNA sequence [3]. Epigenetics is sometimes more narrowly defined as changes that are mitotically and/or meiotically heritable. The molecular turn in epigenetics follows the greater molecular emphasis of biology in general in the latter half of the 20^th^ and the beginning of the twenty-first century [4] and has led to the identification of epigenetic molecular changes including DNA methylation, histone post-translational modifications, non-coding RNA-mediated modifications of chromatin structure, and changes in high-order chromatin structure within the nucleus [5].

Many studies over the last few decades have revealed that these epigenetic states can be influenced by the environment. These studies often fall within the framework of the Developmental Origins of Health and Disease (DOHaD), which emphasizes how early life environmental exposures may condition the health of an individual over their life [6–8]. More surprising is the discovery, in a variety of species, that some environmental insults may have impacts across several generations after direct exposure. This phenomenon is termed transgenerational epigenetic inheritance (TEI) and is the subject of both intense interest and controversy, especially regarding its existence in mammals, including humans [9, 10].

While epidemiological studies in humans can capture associations between environmental triggers and epigenetic changes in easily accessible biospecimen such as blood and urine, and in vitro and rodent models can aid in discovering relevant molecular epigenetic pathways, it is often difficult to compile a complete mechanistic picture that goes from environmental trigger to epigenetic pathway to organismal response using these methods and models. This is especially true for transgenerational experiments, for which the time to generate and handle multiple generations after exposure are long and arduous. The nematode *Caenorhabditis elegans (C. elegans)*, with its quick generation time, well-developed genetic toolkit, ease of handling, and established utility in molecular toxicology has thus emerged as a powerful model organism for mechanistic environmental epigenetic studies [11]. First established as a model organism by Sydney Brenner in the 1970s as part of the effort to bring molecular biology to developmental biology [12], *C. elegans* has played an important role in toxicological research. This is highlighted by early studies examining DNA damage [13] and stress responses [14, 15] in the worm and has expanded over the years to include studies on heavy metal responses [16, 17], toxicogenomic analysis [18], medium-throughput toxicity testing [19], and chemical-induced neurodegeneration [20]. As environmental toxicology has begun to place greater influence on epigenetic mechanisms, so too has the *C. elegans* model. This article will give a brief overview of some fundamental concepts in epigenetics to familiarize outsiders of the field. It will then discuss some of the strengths and weaknesses of *C. elegans* before highlighting some recent key findings (Table 1) that demonstrate its utility as a tractable in vivo system for environmental epigenetics research.

**Table 1:** Select environmental epigenetic studies in *C. elegans*

Manipulation	Epigenetic mark	Main findings	Reference
Genetic manipulations			
*spr-5* (H3K4 demethylase) mutants	6mA	6mA is distributed across the *C. elegans* genome at low levels; NMAD-1 and DAMT-1 regulate 6mA levels; *spr-5* mutants show transgenerational increases in 6mA	Greer et al. 2015 [29]
*spr-5* deletion	H3K4me2	Accumulation of H3K4me2 and progressive decline in fertility and increase in lifespan across generations; H3K4me1/2 and H3K9me3 methyltransferases, an H3K9me3 demethylase, and an H3K9me reader either suppress or accelerate the progressive transgenerational fertility decline	Greer et al. 2014 [58]; Greer et al. 2015 [29]
*ash-2*, *wdr-5*, and *set-2* RNAi	H3K4me3	RNAi against the COMPASS H3K4 methyltransferase complex genes extends lifespan in animals with actively proliferating germlines; reducing germline COMPASS activates a fatty acid desaturation pathway in the intestine that causes accumulation of mono-unsaturated fatty acids sufficient for lifespan extension	Greer et al. 2010 [60]; Han et al. 2017 [61]
*wdr-5*	H3K4me3 and H3K9me2	Transgenerational lifespan extension; global enrichment of H3K9me2; requires the H3K9me2 methyltransferase *met-2*; phenotypic recapitulation by removal of the putative H3K9me2 demethylase *jhdm-1*	Greer et al. 2011 [62]; Lee et al. 2019 [63]
*met-2* mutants	H3K9me2 and small RNAs	Progressive decreases in fertility across generations; requires the Argonaute protein HDRE-1	Lev et al. 2017 [64]
Environmental exposures			
Antimycin (mitochondrial inhibitor)	6mA and H3K4me3	Transgenerational stress adaptation; 6mA levels increase; Increased transcription of mitochondrial stress response genes	Ma et al. 2019 [47]
*Pseudomonas* infection	6mA	Transgenerational stress adaptation; METL-9 is a 6mA methyltransferase; lack of METL-9 lowered induction of innate immune response genes	Ma et al. 2023 [48]
Heat stress	6mA	Transgenerational effects on longevity	Wan et al. 2021 [50]
Antimycin	6mA on mtDNA	mtDNA has higher levels of 6mA than gDNA and increase upon antimycin exposure	Grub et al. 2023 [49]
Methylmercury	H3K4me3	H3K4me3 enrichment in Phase II metabolism genes *lpr-5* and *dpy-7*	Rudgalvyte et al. 2017 [56]
Arsenite, hyperosmosis, starvation	H3K4me3	Adult and transgenerational resistance to hydrogen peroxide requires the H3K4 methyltransferase components *wdr-5.1* and *set-2*	Kishimoto et al. 2017 [57]
Arsenite	H3K4me2	Increased H3K4me2 levels; decreased expression of *spr-5*	Yu and Liao 2016 [35]
Temperature	H3K9me3	Desilencing of a heterochromatic requires the H3K9 methyltransferase *set-25*	Klosin et al. 2017 [66]
Bisphenol A (BPA)	H3K9me3 and H3K27me3	Increases germline apoptosis and embryonic lethality and decreases H3K9me3 and H3K27me3 up to the F3 generation; rescued by targeting the Jumonji demethylases JMJD-2 and UTX-1	Camacho et al. 2018 [30]
Ethanol	Unknown	Transgenerational increases in apoptosis, aneuploidy, and embryonic lethality; single-nucleus RNA sequencing revealed cell and tissue specific responses across generations	Truong et al. 2023 [67]
*Pseudomonas aeruginosa* or *P. vranovensis* infection	Small RNAs, H3K4me3, and H3K9me3	Inheritance of learned avoidance for four generations; parental avoidance requires *set-2*, *wdr-5.1*, *rbr-2*, and *set-32*; F1 avoidance requires *set-25* and *hpl-2*; transgenerational avoidance requires TGF- ß and the Piwi Argonaute small RNA pathway; the bacterial small RNAs P11 or Pv1 are sufficient to induce transgenerational learned avoidance; learned avoidance can be passed to naïve animals via *Cer1* particles	Moore et al. 2019 [68]; Kaletsky et al. 2020 [70]; Moore et al. 2021 [71]; Sengupta et al. 2023 [72]

## Fundamental Concepts in Epigenetics

- **Epigenetics**: Originally described the process in which genetic variations give rise to distinct patterns of cellular differentiation. Modern usage broadly refers to molecular mechanisms that regulate gene expression and subsequent phenotypes in the absence of changes in DNA sequence. Sometimes epigenetics refers only to the mechanisms that lead to mitotically or meiotically heritable phenotypes. The most-studied epigenetic mechanisms of gene regulation are the covalent modifications of DNA and histone proteins which induce chromatin structural changes that influence DNA–protein interactions and subsequent gene expression, though regulatory RNAs also play an important epigenetic role.
- **Epigenome**: The totality of epigenetic modifications found in a cell, tissue, or organism. Exists as an equilibrium that is influenced by the cellular, tissue, or organismal environment.
- **Chromatin**: The mixture of DNA and histone proteins that makes up the chromosomes in cells. Chromatin generally comes in two varieties: euchromatin, which is relaxed and accessible to transcription proteins, and heterochromatin, which is largely condensed and silent.
- **Histones**: Proteins found in the nucleus of all eukaryotes that organize DNA. In most cases, two of each of the four core histones – H2A, H2B, H3, and H4 – form an octamer which DNA then wraps around to form a nucleosome. Histone H1 is a linker protein between nucleosomes.
- **Cytosine methylation**: Cytosine methylation occurs by enzymatic transfer of a methyl group to carbon 5 of the pyrimidine base cytosine (5-methylcytosine or 5mC) in the 5’-3’ cytosine guanine (CpG) dinucleotide sequence. 5mC is a common epigenetic mark in mammals and lack of 5mC in *C. elegans* is therefore one of the major drawbacks to using this model organism for epigenetics.
- **CpG island**: A region of genomic DNA with a minimum length of 200 bp, unusually high cytosine and guanine (CG) levels, and a high frequency of CpG dinucleotides. Usually found in the regulatory regions of genes near promoters, CpG islands are the primary target for DNA methyltransferases.
- **Adenine methylation**: Enzymatic transfer of a methyl group to nitrogen 6 of the purine base adenine (6-methyladenine or 6mA). While originally thought to only exist in prokaryotes, 6mA has been reported in the DNA of eukaryotes ranging from multicellular plants to mammals including humans, although its existence in more recently evolved eukaryotes remains a topic of debate.
- **DNA methyltransferases (DNMTs):** Enzymes that catalyze the transfer of a methyl group to cytosine to form 5mC or adenine to form 6mA. In mammals four isoforms of cytosine DNMTs exist, while *C. elegans* lacks any homologs. The identity of adenine methyltransferases is under investigation.
- **Histone post-translational modification (PTM):** A covalent modification of a histone protein that can alter gene expression positively or negatively depending on the modification, which amino acid residue is modified, and its position relative to gene regulatory regions. Most commonly refers to methylation and acetylation, though other PTMs also exist, including phosphorylation, ubiquitination, sumoylation, acylation, lactylation, and neurotransmitter-based modifications.
- **Non-coding RNA:** Regulatory RNAs that are not transcribed. This includes small RNAs such as micro (mi), small interfering (si), and PIWI-interacting (pi) RNAs and long non-coding (lnc) RNAs.

### Strengths and Limitations of *C. Elegans* as a Model for Environmental Epigenetics

Critical evaluation of the characteristics of *C. elegans* that make it suitable as a model for environmental epigenetics has been carried out elsewhere [11] and will be briefly summarized here. One of the greatest advantages of using *C. elegans* is its short life cycle (3–4-day generation time), which allows for efficient developmental, aging, and transgenerational experiments. *C. elegans* was also the first metazoan genome to be sequenced, with around 70% of its genes having human homologs [21]. This, combined with powerful genetic tools such as gene knockout and RNA interference (RNAi) libraries, widely available transgenic strains, and CRISPR-dCas9 editing, has made *C. elegans* a mainstay in genetic studies since its introduction in the 1970s. *C. elegans* has the additional advantages of a transparent body, an invariant somatic developmental pattern and anatomy, and stereotyped locomotion and behavioral responses to the environment.

Importantly for environmental epigenetics, testing chemical toxicity in *C. elegans* seems to be nearly as predictive as the standard rat model, as evidenced by a high throughput toxicity screening of nearly 1,000 chemicals by the National Toxicology Program [22]. This is thought to be due to conservation of molecular targets and signaling pathways important for responses to chemical toxicants. Thanks to efforts such as the modENCODE project, we also now know that, though there are specific examples of differences between species, there is broad conservation of chromatin regulation between *C. elegans* and humans [23]. This includes conservation of core histone proteins, chromatin remodeling enzymes, and patterns of histone modifications.

While *C. elegans* is a powerful model for molecular environmental epigenetics research, it does have a few obvious limitations that should be taken into careful consideration. For example, one aspect to consider is the lack of some mammalian anatomical features, including certain cell types such as blood and immune cells, and organ systems that are relevant to chemical exposure and metabolism in mammals such as lungs, kidneys, and a blood brain barrier [11]. Phenomena that are only found in mammalian cellular environments, and cell-type specific epigenetic mechanisms in cells absent from *C. elegans*, are impossible to model in the nematode. Lack of blood and immune cells is a point of concern as human epigenetic studies are often limited to these readily accessible biospecimen, making cross-species tissue to tissue epigenetics comparisons difficult. Furthermore, there are key genetic differences that hamper interpretation of experiments meant to unveil environmental epigenetic molecular mechanisms. Two important ones are differences in metabolic enzymes, including in the toxicologically important cytochrome P450s [24], and lack of the DNA methylation mark 5mC [25]. *C. elegans* has a larger complement of cytochrome P450 genes than humans (76 vs 57) and can metabolize many xenobiotics although P450s of family 1 may be missing in the nematode [26, 27]. So-called “humanized” strains that introduce human P450s could be used to overcome this although the higher number of P450s present and the lack of characterization of their substrate may complicate result interpretation.

Another difference between *C. elegans* and mammalian epigenetic models is its lack of genomic 5mC. This is a weakness shared by other commonly used invertebrate model organisms, including yeast and *Drosophila*. While many invertebrates display mosaic methylation, or regions of methylated DNA interspersed with unmethylated domains, *C. elegans* has undetectable levels of 5mC [28]. *C. elegans* also lacks homologs of the DNA methylation enzymes DNA (cytosine-5-)-methyltransferase 1 (DNMT1) and DNMT3, lending further evidence to the view that *C. elegans* does not actively have 5mC methylation [29]. Lack of 5mC in *C. elegans* can be an advantage, however, as it allows researchers to isolate the effects of environmental stressors on other epigenetic marks, such as the evolutionary conserved histone methylation marks, and investigate their role in epigenetic inheritance [30]. Additionally, the presence of a different DNA methylation mark, 6mA, and its role in responses to environmental stressors has been the target of recent investigation and will be discussed below. Despite the lack of 5mC and metabolic differences between humans and *C. elegans*, responses to environmental chemicals can provoke the same epigenetic changes Arsenic exposure, for example, leads to increased levels of H3K4me2 and H3K4me3 in a variety of settings: in vitro exposure of a human lung cancer cell line [31], in the leukocytes of steel workers who had inhaled arsenic-contaminated particulate matter [32], in the peripheral blood mononuclear cells of Bangladeshi adult women exposed to arsenic in water [33], in the dentate gyrus and frontal cortex of male mice [34], and in *C. elegans* across multiple generations [35]. While these findings suggest that these histone PTMs could be used as a general biomarker of arsenic exposure, caution must be exercised in their interpretation as the Bangladesh and mouse brain studies showed sex-specific H3K4 methylation changes.

### What is Transgenerational Epigenetic Inheritance?

**Fig. 1:**
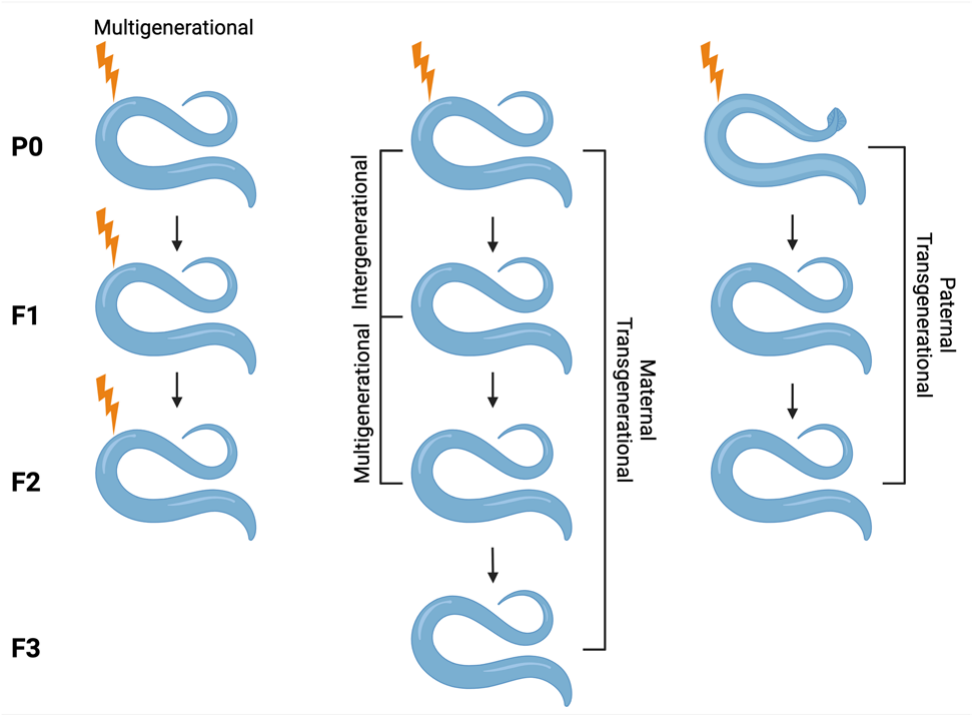
Inter-, multi-, and transgenerational inheritance. The primary difference between these three modes of inheritance is the timing/duration of environmental exposure (lightning bolt) across generations (P0, F1-F3). Intergenerational inheritance refers to a parental exposure (P0) causing changes in the direct offspring (F1) who were also directly exposed in utero. Multigenerational inheritance refers either to exposure to environmentally persistent stressors (left column), or to changes in the F2 generation after maternal P0 exposure. Since the germ cells that give rise to the F2 offspring are present during a P0 maternal exposure, these animals are also directly exposed. Transgenerational inheritance refers to changes in the F3 generation after maternal P0 exposure or F2 generation after paternal P0 exposure. These animals are the first to have no direct exposure

One of the most powerful aspects of using *C. elegans* as a model for environmental epigenetics is the ability to quickly perform transgenerational epigenetic inheritance (TEI) experiments with robust in vivo mechanistic analyses. Growing evidence suggests that inheritance across generations occurs in mammals, though it remains controversial, especially in humans [9, 10]. Inheritance of non-DNA sequence-based epigenetic information in invertebrates [36] and plants [37] is much more accepted, with *C. elegans* leading the charge in transgenerational studies. Specific recent findings will be discussed in subsequent sections, but it is important at the outset to describe what TEI is and how it differs from intergenerational or multigenerational inheritance (Fig. 1). Intergenerational inheritance refers to the passage of epigenetic information from the parental generation (P0) to the first-generation (F1) offspring. Within an environmental exposure paradigm, because the germ cells that give rise to the F1 generation are present during any P0 exposure, the F1 generation is also directly exposed. Multigenerational exposure usually refers to one of two scenarios: either maternal passage to the second generation (F2) offspring or exposures that persist through multiple generations. In the first case, the F2 generation is still directly exposed since the F1 generation also contains the germ cells that will give rise to the F2 generation. The latter case is exemplified by environmentally persistent chemicals such as the pesticide dichloro-diphenyl-trichloroethane (DDT) [38].

True TEI involves maternal transmission of epigenetic information to the third (F3) generation or paternal transmission to the F2 generation. This information must be passed via molecular signals and can either be replicated across generations or reconstructed in the progeny via secondary signals [39]. Replicative transmission takes place via DNA methylation and/or histone modification and involves maintenance of these marks through the genome-wide reprogramming, or erasure, that occurs during gametogenesis and fertilization. Maintenance of environmentally-induced epigenetic changes through these reprogramming events is debated in mammals, although increasing evidence suggests that environmental chemicals can influence the regulatory mechanisms of epigenetic marks. Reconstruction of DNA methylation and histone modification sites after germline and embryonic reprogramming can either be genetic (driven by transcription factor recognition of specific sequences) or epigenetic. Non-coding RNAs are among the most well-studied epigenetic reconstruction signal, with much of the general mechanisms worked out in *C. elegans* studies [40–42] along with compelling evidence of a role for tRNA-derived small RNAs in intergenerational inheritance in mice and humans [43, 44].

### DNA Methylation

Though *C. elegans* lacks the key DNA methylation mark 5mC, the presence of 6mA in the *C. elegans* genome has recently gained attention. While originally thought to be unique to prokaryotes, more recent evidence points to a role of 6mA among multicellular eukaryotes [45], including in humans and diseases such as cancer [46]. Eukaryotic 6mA needs further confirmation before it can be said to be a bona fide epigenetic mark (see below), with studies in *C. elegans* aiding this effort. In the nematode, 6mA was found to be distributed across the genome at a low level (~ 0.01% of adenines) [29], though still higher than the generally accepted level in humans (1–3 parts per million) [45]. Active 6mA regulators were also discovered, including a 6mA DNA demethylase (NMAD-1) and a methyltransferase (DAMT-1). Interestingly, mutants lacking the *C. elegans* histone H3 lysine 4 dimethyl demethylase, *spr-5*, showed transgenerational increases in 6mA (see below for a longer discussion of *spr-5* and its role in transgenerational longevity). This points to a possible connection between 6mA regulation and histone methylation.

Recent work has begun to reveal the biological function of 6mA, including roles in stress responses, immunity, and mitochondrial genome regulation [47–49]. Exposure to mitochondrial inhibitors such as antimycin leads to developmental delays in *C. elegans*; however, the progeny of exposed parents showed significant stress adaptation [47]. Remarkably, this adaptation lasted multiple generations (up to F4) after a single parental exposure, suggesting transgenerational inheritance of mitochondrial stress adaptation. Histone H3K4me3 and DNA 6mA modifications seem to be required for transmission to progeny, with 6mA levels also increasing upon mitochondrial perturbation. Elevated 6mA following mitochondrial inhibition is mostly due to increased expression on mitochondrial stress response genes, promoting their transcription to alleviate stress in the progeny. Importantly, a *Pseudomonas* bacterial strain that infects *C. elegans* and activates the mitochondrial stress response also leads to a transgenerational adaptive response. A subsequent study from the same lab confirmed that 6mA levels change upon pathogenic bacterial infection and identified METL-9 as the 6mA methyltransferase [48]. Lack of METL-9 led to lower induction of innate immune response genes and made animals more susceptible to pathogen infection. Heat stress in *C. elegans* also leads to 6mA-regulated transgenerational effects on longevity [50] and so it is possible that other environmental stressors, such as the mitochondrial electron transport chain inhibitor rotenone, could influence 6mA-regulated transgenerational adaptations in *C. elegans*. Interestingly, chronic stress in mice leads to the accumulation of 6mA in the pre-frontal cortex [51], pointing to shared responses to environmental stressors across species and the potential to uncover regulatory mechanisms in *C. elegans* that are conserved in mammals. Indeed, two recent studies, one in a human cell line and one in *C. elegans,* have shown that mitochondrial DNA (mtDNA) contains higher levels of 6mA than genomic DNA does, and that levels are regulated by environmental stressors. The first study was done in the human liver cancer cell line HepG2 under hypoxic conditions [52] while the second showed that *C. elegans* mtDNA is dynamically regulated in response to antimycin [49].

These studies represent an exciting new avenue for research into 6mA as a conserved, environmentally influenced regulatory mark; however, further experiments will need to be done to confirm that 6mA is a directed epigenetic modification in multicellular eukaryotes with real biological functions [45]. The low levels of 6mA detected in eukaryotic DNA means that detection experiments are highly susceptible to both artefacts and contamination of samples. This is especially a concern for researchers using the bacterivore *C. elegans*, where bacteria lining the intestine could lead to erroneous measurements of 6mA levels. There is also the possibility that genomic 6mA is due to incorporation of pre-methylated bases from RNA or foreign DNA via nucleotide salvage pathways [53]. This possibility could be distinguished from 6mA as a directed modification as incorporation of bases would lead to a random distribution while directed modification would lead to a consistent distribution in the genome under the same experimental conditions. There is also the concern that the putative 6mA regulatory enzymes identified thus far may not have specificity or preference for DNA over alternative substrates such as small nuclear RNAs, which would make 6mA on DNA physiologically irrelevant [54]. More thorough in vitro and in vivo experiments into the kinetics of 6mA methylases and demethylases need to be conducted to confirm physiologically relevant activity. The evidence of 6mA on mtDNA is more convincing, both because of the higher levels detected and the ancient origins of mitochondria making it more likely that a regulatory role has been retained in the organelle, but still need to be confirmed. Going forward, epigenomic editing experiments targeting 6mA at specific genes will allow for demonstration of causal roles for 6mA. This, along with multiple other independent techniques will need to be used to confirm the existence and dynamics of 6mA before it can definitively be said to be a bona fide epigenetic mark in multicellular eukaryotes.

### Histone Post-translational Modifications and Non-coding RNA

The conservation of histone proteins and histone regulatory pathways between *C. elegans* and humans means that much of the work done with *C. elegans* in environmental epigenetics has focused on environmental stressors that influence histone PTMs and their interaction with non-coding RNAs. Some past, key studies have been discussed in depth elsewhere [11, 55] and so will only be briefly summarized here before going into more depth on a few recent findings. Methylmercury [56], arsenite [35, 57], hyperosmosis [57], and starvation [57] have all been found to increase the active marks H3K4me2 or H3K4me3. Exposure to any of the latter three stressors during development seems to increase both adult and transgenerational resistance to hydrogen peroxide and requires the H3K4 methyltransferase components *wdr-5.1* and *set-2* [57]. Arsenite may also lead to increased H3K4me2 levels for up to three generations via reduced expression of the H3K4me2 demethylase *spr-5* [35].

As mentioned above, deletion of *spr-5* itself leads to inherited accumulation of H3K4me2 and progressive decline in fertility [58], which may explain the transgenerational reproductive effects of arsenite. The progressive transgenerational phenotype in *spr-5* mutants depends upon a network of chromatin regulators that can control the flow of epigenetic information across generations, including H3K4me1/2 and H3K9me3 methyltransferases, an H3K9me3 demethylase, and an H3K9me reader. Interestingly, *spr-5* deletion also causes a transgenerational increase in lifespan via activation of a hormonal signaling pathway known to regulate lifespan [59], suggesting a that there is a transgenerational tradeoff between fertility and lifespan that can be regulated by histone methylation and germline-to-soma transmission. Indeed, animals with reductions in the COMPASS H3K4 methyltransferase complex genes *wdr-5, ash-2*, and *set-2* live longer than wild-type individuals as long as an actively proliferating germline is present [60]. A follow up study found that reducing COMPASS solely in the germline activates a fatty acid desaturation pathway in the intestine that increases lifespan, and that activation of this pathway causes the accumulation of mono-unsaturated fatty acids that is sufficient to extend lifespan [61].

The H3K4 methyltransferase *wdr-5* mutant longevity phenotype is heritable across multiple generations [62]. Intriguingly, a recent study has demonstrated that this transgenerational longevity corresponds with global enrichment of H3K9me2 over 20 generations, requires the H3K9me2 methyltransferase MET-2, and can be recapitulated by removal of the H3K9me2 demethylase *jhdm-1* [63]. A separate study on H3K9 methylation levels highlighted the interplay between post-translational histone modifications and non-coding RNAs: mutants for the H3K9 methyltransferase *met-2* themselves show progressive decreases in fertility over 30 generations [64]. This progressive sterility phenotype requires the Argonaute protein HDRE-1, which had previously been shown to carry small RNAs across generations to drive multigenerational RNAi inheritance [65]. While normally heritable for a few generations in *C. elegans*, *met-2* mutants show persistence of RNAi up to 15 generations [64]. This suggests that MET-2 controls the production of small RNAs via regulation of H3K9 methylation, demonstrating another possible connection between different types of epigenetic mechanisms, and expanding the network of epigenetic regulation to include multiple histone methylation sites interacting with small RNAs. How many generations these transgenerational progressive phenotypes in mutants persist remains to be seen, although the progressive increase in effect size over generations suggests that the phenotypes persist for longer than what has been observed thus far.

H3K9 methylation is also sensitive to environmental stressors. High temperatures seem to decrease the repressive mark H3K9me3 for 14 generations, an effect which requires the H3K9 methyltransferase *set-25* [66]. A more recent study extends the role of H3K9me3, as well as H3K27me3, to the transgenerational effects of Bisphenol A (BPA) [30]. Parental BPA exposure causes an increase in germline apoptosis and embryonic lethality and a reduction in the repressive marks H3K9me3 and H3K27me3 up to the F3 generation. Importantly, targeting the Jumonji demethylases JMJD-2 and UTX-1 restores H3K9me3 and H3K27me3 levels and alleviates the BPA-induced effects. Whether small RNA-based mechanisms are also involved in the response to BPA remains unclear, though it seems likely given the evidence of a connection in the context of other types of stressors as described above. A transgenerational response to alcohol was also recently described involving increases in apoptosis, aneuploidy, and embryonic lethality [67]. Utilizing single-nucleus RNA sequencing, a tissue and cell-type resolution transcriptome was established across generations after parental ethanol exposure. Although the epigenetic mechanism responsible for the transgenerational response and transcriptomic differences remains to be determined, this approach shows the promise of using modern sequencing techniques and the *C. elegans* model to tackle problems in environmental epigenetics.

One of the most exciting recent findings, and one that ties together both RNA-based and histone-based mechanisms, was the discovery that *C. elegans* can inherit learned avoidance of the pathogenic bacteria *P. aeruginosa* (strain PA14) for four generations [68]. It has long been known that while *C. elegans* is initially attracted to *P. aeruginosa*, it learns to avoid it within hours of exposure [69]. Surprisingly, this learned avoidance can be passed down to progeny for up to four generations via transforming growth factor ß (TGF- ß) signaling in sensory neurons and the Piwi Argonaute small RNA pathway. While mutants for the H3K4me3 methyltransferases (*set-2* and *wdr-5.1*), the H3K4me3 demethylase *rbr-2*, and the H3K9me3 methyltransferase *set-32* were already defective in naïve P0 attraction to PA14 and in aversive pathogenic learning, meaning their role in transgenerational inheritance could not be determined, the H3K9 methyltransferase *set-25* and the H3K9me3 reader *hpl-2* were found to have normal P0 naïve attraction and learned avoidance of *P. aeruginosa* but were defective in F1 avoidance of *P. aeruginosa*. These two genes function downstream of the nuclear RNAi pathway, suggesting a link between histone PTMs and non-coding RNAs in learned pathogen avoidance. In a follow up study, the Murphy lab showed that a single exposure to a purified small RNA isolated from *P. aeruginosa*, P11, was sufficient to induce transgenerational learned pathogen avoidance via the same small RNA pathways [70]. Even more surprisingly, the memory of encountering this bacterial small RNA can be passed from infected to naïve animals via *Cer1* retrotransposon-encoded virus-like particles [71]. *Cer1* also seems to function internally to transmit information from the germline to the neurons to allow the nematode to execute avoidance behaviors via reduction of a key chemotaxis neuronal gene, *maco-1*. These studies were done using a clinical isolate of *P. aeruginosa*, PA14, that *C. elegans* would not encounter in its natural habitat; however, a recent preprint from the Murphy lab suggests that a small RNA from a *Pseudomonas vranovensis* strain isolated from the *C. elegans* microbiota, Pv1, also induces transgenerational learned avoidance, suggesting that transgenerational inheritance is a natural part of the *C. elegans* defense response [72].

The findings with *Cer1* complement emerging work in *C. elegans* that suggests that germ granules, germline liquid-like condensates that concentrate components of the siRNA machinery, are crucial coordinators of epigenetic inheritance pathways [73, 74]. What’s more, a recent report showed that loss of *eggd-1*, a LOTUS domain protein crucial for the formation of perinuclear germ granules in *C. elegans*, leads to activation of an HLH-30-mediated transcriptional program in somatic tissues, demonstrating a germ granule-to-soma communication pathway [75]. The packaging and concentration of small RNAs thus represents yet another avenue for environmental influence on the epigenome that can have impacts across multiple generations. It should be noted that these environmentally induced transgenerational phenotypes seem to be transient, with effects lasting only 3–5 generations and effect sizes shrinking with each subsequent generation. This is different than the progressive transgenerational phenotypes caused by mutations in epigenetic regulatory genes, and the mechanisms responsible for ending the environmentally-induced transgenerational response remains to be determined.

## Conclusions

The studies highlighted above demonstrate that multiple different environmental stressors can influence various, often overlapping, epigenetic pathways. These pathways often function together to create a network of epigenetic regulation involving multiple sites of histone methylation and small RNAs that govern responses to environmental stressors across generations. Going forward, it will be crucial to take advantage of modern sequencing technologies and multiomics approaches to get a larger, unbiased view of the epigenomic network responses to environmental stressors. For example, following an exposure quantitation of large numbers of histone PTMs using mass spectrometry can be used to screen for relevant PTMs [76]. This can be followed up by CUT&TAG to look for locus-specific changes in histone PTMs [77] and RNA-seq to look for corresponding transcriptional changes and to profile small RNAs [78]. Clear definitions of inter-, multi-, and trans-generational inheritance will aid analysis and interpretation, as each mode of inheritance likely arise from different mechanisms [79]. Along these lines, it will also be important to distinguish between mechanisms responsible for initiating, maintaining/transmitting, and terminating epigenetic inheritance. Carrying these experiments out in *C. elegans* will allow for efficient discovery of conserved epigenetic pathways that play a role in responses to the environment across development and generations.

## Key References

- Breton CV, Landon R, Kahn LG, Enlow MB, Peterson AK, Bastain T, et al. Exploring the evidence for epigenetic regulation of environmental influences on child health across generations. Commun Biol 2021;4:769. 10.1038/s42003-021-02316-6
  - ○ A critical evaluation of the evidence for transgenerational epigenetic inheritance in humans.
- Fitz-James MH, Cavalli G. Molecular mechanisms of transgenerational epigenetic inheritance. Nat Rev Genet 2022;23:325–41. 10.1038/s41576-021-00438-5
  - ○ A thorough overview of the many possible routes of transgenerational inheritance.
- Boulias K, Greer EL. Means, mechanisms and consequences of adenine methylation in DNA. Nat Rev Genet 2022;23:411–28. 10.1038/s41576-022-00456-x
  - ○ A critical evaluation of the evidence for adenine methylation in eukaryotes.
- Ma C, Xue T, Peng Q, Zhang J, Guan J, Ding W, et al. A novel N6-Deoxyadenine methyltransferase METL-9 modulates C. elegans immunity via dichotomous mechanisms. Cell Res 2023;33:628–39. 10.1038/s41422-023-00826-y
  - ○ Presents compelling evidence for 6mA playing a role in transcriptional regulation of innate immunity upon pathogen infection.
- Moore RS, Kaletsky R, Lesnik C, Cota V, Blackman E, Parsons LR, et al. The role of the Cer1 transposon in horizontal transfer of transgenerational memory. Cell 2021;184:4697-4712.e18. 10.1016/j.cell.2021.07.022
  - ○ The latest in a series of published articles uncovering a detailed mechanism for transgenerational inheritance of learned pathogen avoidance.
- Price IF, Wagner JA, Pastore B, Hertz HL, Tang W. C. elegans germ granules sculpt both germline and somatic RNAome. Nat Commun 2023;14:5965. 10.1038/s41467-023-41556-4
  - ○ An elegant study demonstrating the importance of germ granules in mediating a transcriptional program in somatic tissues.

## Data Availability

No datasets were generated or analysed during the current study.
